# Supplementary material for: Resolving the full spectrum of human genome variation using Linked-Reads
Source: Genome Res. 2019 Apr;29(4):635–45. doi: 10.1101/gr.234443.118 (PMC6442396; doi:10.1101/gr.234443.118)
Supplement: Supplemental Material [file supp_gr.234443.118_Supplemental_longranger-2.2.zip › longranger-2.2.tar.bz2!/lr2.2-public/lib/stan/cmdstan/stan/doc/api/html/arg__adapt_8hpp_source.html]

Stan: src/stan/gm/arguments/arg\_adapt.hpp Source File


|  |  |
| --- | --- |
|  | Stan  2.5.0  probability, sampling & optimization |


- Main Page
- Related Pages
- Namespaces
- Classes
- Files

- File List
- File Members

All Classes Namespaces Files Functions Variables Typedefs Enumerations Enumerator Friends Macros Pages

- src
- stan
- gm
- arguments

arg\_adapt.hpp

Go to the documentation of this file.

1 #ifndef STAN\_\_GM\_\_ARGUMENTS\_\_ADAPT\_\_HPP

2 #define STAN\_\_GM\_\_ARGUMENTS\_\_ADAPT\_\_HPP

3

4 #include <stan/gm/arguments/categorical\_argument.hpp>

5

6 #include <stan/gm/arguments/arg\_adapt\_engaged.hpp>

7 #include <stan/gm/arguments/arg\_adapt\_gamma.hpp>

8 #include <stan/gm/arguments/arg\_adapt\_delta.hpp>

9 #include <stan/gm/arguments/arg\_adapt\_kappa.hpp>

10 #include <stan/gm/arguments/arg\_adapt\_t0.hpp>

11 #include <stan/gm/arguments/arg\_adapt\_init\_buffer.hpp>

12 #include <stan/gm/arguments/arg\_adapt\_term\_buffer.hpp>

13 #include <stan/gm/arguments/arg\_adapt\_window.hpp>

14

15 namespace stan {

16

17  namespace gm {

18

19  class arg\_adapt: public categorical\_argument {

20

21  public:

22

23  arg\_adapt() {

24

25  \_name = "adapt";

26  \_description = "Warmup Adaptation";

27

28  \_subarguments.push\_back(new arg\_adapt\_engaged());

29  \_subarguments.push\_back(new arg\_adapt\_gamma());

30  \_subarguments.push\_back(new arg\_adapt\_delta());

31  \_subarguments.push\_back(new arg\_adapt\_kappa());

32  \_subarguments.push\_back(new arg\_adapt\_t0());

33  \_subarguments.push\_back(new arg\_adapt\_init\_buffer());

34  \_subarguments.push\_back(new arg\_adapt\_term\_buffer());

35  \_subarguments.push\_back(new arg\_adapt\_window());

36

37  }

38

39  };

40

41  } // gm

42

43 } // stan

44

45 #endif

46

stan::gm::arg\_adapt\_gamma

**Definition:** arg\_adapt\_gamma.hpp:10

arg\_adapt\_delta.hpp

stan::gm::arg\_adapt\_t0

**Definition:** arg\_adapt\_t0.hpp:10

arg\_adapt\_window.hpp

categorical\_argument.hpp

stan::gm::arg\_adapt\_engaged

**Definition:** arg\_adapt\_engaged.hpp:10

stan::gm::categorical\_argument

**Definition:** categorical\_argument.hpp:11

stan::gm::categorical\_argument::\_subarguments

std::vector< argument \* > \_subarguments

**Definition:** categorical\_argument.hpp:153

arg\_adapt\_engaged.hpp

stan::gm::arg\_adapt::arg\_adapt

arg\_adapt()

**Definition:** arg\_adapt.hpp:23

arg\_adapt\_term\_buffer.hpp

arg\_adapt\_gamma.hpp

stan::gm::arg\_adapt\_kappa

**Definition:** arg\_adapt\_kappa.hpp:10

arg\_adapt\_t0.hpp

stan::gm::argument::\_name

std::string \_name

**Definition:** argument.hpp:76

stan::gm::arg\_adapt\_term\_buffer

**Definition:** arg\_adapt\_term\_buffer.hpp:10

arg\_adapt\_kappa.hpp

stan::gm::arg\_adapt\_init\_buffer

**Definition:** arg\_adapt\_init\_buffer.hpp:10

stan::gm::arg\_adapt

**Definition:** arg\_adapt.hpp:19

stan::gm::arg\_adapt\_window

**Definition:** arg\_adapt\_window.hpp:10

stan::gm::arg\_adapt\_delta

**Definition:** arg\_adapt\_delta.hpp:10

arg\_adapt\_init\_buffer.hpp

stan::gm::argument::\_description

std::string \_description

**Definition:** argument.hpp:77


---

[ Stan Home Page ]

*© 2011–2014,
Stan Development Team.*
